# Students’ Motivational and Emotional Experiences in Physical Education across Profiles of Extracurricular Physical Activity: The Influence in the Intention to Be Active

**DOI:** 10.3390/ijerph19159539

**Published:** 2022-08-03

**Authors:** Sebastián Fierro-Suero, Eduardo José Fernández-Ozcorta, Pedro Sáenz-López

**Affiliations:** 1 Faculty of Education, Psychology and Sport Sciences, Universidad de Huelva, Avda. Tres de marzo s/n, 21071 Huelva, Spain; psaenz@uhu.es; 2 Department of Physical Activity and Sports, Center for University Studies Cardenal Spínola CEU, University of Seville Attached Centre-Spain, 41930 Bormujos, Spain

**Keywords:** intention to be physically active, basic psychological needs, autonomous motivation, control-value theory, self-determination

## Abstract

This study examined the relationship between extracurricular physical activity (PA) levels and students’ motivational and emotional experience during physical education (PE) classes and how this psychological experience can predict the intention to be physically active. The sample consisted of 811 Spanish secondary education students (371 boys and 440 girls) aged between 11 and 17 years (M = 13.15, SD = 1.16). Students completed questionnaires about their PA levels, their intention to be physically active, and their motivational and emotional experience during PE classes. A cluster analysis was used to classify the students according to their level of extracurricular PA. Based on a regression analysis, the variables enjoyment, pride, hopelessness, competence, satisfaction, and autonomous motivation played the highest role, predicting the intention to be physically active in the future. Statistical differences were found among the different PA profiles in these variables during the PE classes (MANCOVA). In conclusion, hours of PA outside school have a high relationship with the students’ emotional and motivational experience in their PE classes, which is related with the intention to practise PA in the future. A series of strategies have been proposed at both the institutional level and the teacher level to improve the PE psychological experience of those students who practise less extracurricular PA.

## 1. Introduction

It is indisputable that behaviours that limit the amount of physical activity (PA) performed are entrenched in society [1], even if they are combated by recommendations at the supranational level such as the World Health Organization (WHO) [2]. In this debate, children and adolescents are in the spotlight, given that sedentary and inactive behaviours seem to continue from childhood through adolescence into adulthood [3] and, at present, data indicate that more than three quarters of adolescents (81%) do not meet the recommendations for aerobic exercise [4] and 55.4% of young Spaniards are sedentary in their out-of-school time [5].

Specifically, we know that regular PA, especially of moderate to vigorous intensity, has been consistently associated with indicators of physical health [6], psychological health [7], and even optimal growth—maturation as well as physical [8] and cognitive development [9]. Along these lines, data reveal that many children and adolescents in developed countries have sedentary lifestyles [7], with fewer active leisure activities and a greater reliance on sedentary lifestyles [4].

Currently, the WHO [2] international PA recommendations for children and adolescents are for an average of at least 60 min/day of moderate to vigorous intensity PA, mainly aerobic, during the week. These action recommendations are coupled with limiting the amount of time spent in sedentary activities, particularly the amount of time using/viewing recreational screens [10]. Therefore, taking these recommendations into account, the data on the PA practised during the time spent in school indicate that recess and PE classes of the student body do not, for the most part, comply with the recommendations [11].

To address this problem, firstly, adolescents should perform more PA during the school schedule [11], with physical education (PE) playing an important role in the promotion of PA [12]. However, according to De Meester et al. [13], there is a limit to the curriculum time devoted to PE, being different according to the geographical area in which one is located. For example, in Poland, Austria, and Hungary, PE hours are around 10–11% of the total teaching hours. However, in other countries such as Spain, Malta, and Turkey, it is only 3–4% [14]. Additionally, PE addresses a wide variety of topics, not all of which can be developed with high levels of physical practice. For these reasons, it may be necessary to explore possible additional sources of PA opportunities, including extracurricular PA participation [15]. In turn, as the literature has shown [16,17,18], positive psychological conditions during PE have been proposed as positive predictors of the intention to engage in physical activity. The intention to be physically active has been widely used in the literature as a predictor of the practice of physical activity [19,20,21,22], since it is a determinant of active behaviour [22,23]. Knowledge of intentions starts from socio-cognitive theories, such as the theory of planned behaviour [24]. These theories aim to explain behaviour through socio-cognitive factors. Specifically, intentions are produced by favourable evaluations of a behaviour (attitudes), perceptions that others expect such behaviour (subjective norms), and beliefs that the behaviour is under the person’s control (perceived behavioural control). Understanding this perspective can explain why some people have stronger intentions than others. However, there are studies in which intention has not been linked to the practice of PA [25], but rather it has been shown that other complementary constructs are necessary to improve the ability to predict PA [26]. To satisfy this requirement, other approaches have been proposed to extend the theory by disaggregating motivation [27] and emotions [18].

### 1.1. Self-Determination Theory

In terms of motivation, one of the most important theories is the self-determination theory (SDT). According to this theory, motivation shows a continuum that ranges from amotivation, through extrinsic motivation to intrinsic motivation [28,29]. Amotivation is the lack of intention to take part. Extrinsic motivation is divided into four types of regulation depending on the external focus which is the reason for deciding to be part of the activity: external, introjected, identified, and integrated regulation. Finally, intrinsic motivation is when people take part because of their enjoyment of the activity. This is the most self-determined type and is related to positive behavioural consequences such as the intention to be active in the future [17] or academic achievement in PE [30]. According to this theory, self-determined motivation is influenced by three basic psychological needs (BPNs): autonomy, competence, and relatedness [31]. Autonomy is the need to feel that one is key to one’s own experiences and actions. Competence refers to feeling effective when undertaking the behaviour. Relatedness refers to feeling socially connected to others. The more the students’ basic needs are satisfied, the more self-determined motivation may show, leading to change in exercise behaviours [32].

### 1.2. Control-Value Theory of Achievement Emotions

Different emotional experiences are established through appraisal of the environment and of oneself and one’s conceptual knowledge [33]. For this, the Control-Value Theory of Achievement Emotions (CVTAE) [34] has been used in various subjects in education such as PE [18,35,36,37]. Achievement emotions can be defined as emotions that are related directly to achievement activities such as physical sport activities and achievement outcomes (success and failure). The CVTAE classifies emotions in three dimensions: valence (positive or negative), activity level (activating or deactivating), and object focus (activity or outcome). Within this classification, emotions act uniquely in each of the proposed dimensions. Enjoyment is a positive, activating emotion focused on the activity; pride is a positive, activating emotion focused on the outcome; boredom is a negative, deactivating emotion focused on activity; hopelessness is a negative, deactivating emotion focused on the outcome; anxiety is a negative, activating emotion focused on the outcome; and anger is a negative, activating emotion. Emotions and intentions to engage in PA have been less studied, although relationships between emotional experiences and levels of PA are beginning to be elucidated [18]. In this regard, the literature has reported, for example, that enjoyment is associated with higher levels of PA [18,38] and higher intention to practise it in the future [39], whereas boredom is associated with lower levels of PA [36,40]. Other emotions, such as anger experienced during PE class, appear to be unrelated to sedentary behaviour outside school [18,36]. As can be seen, emotional experience seems to condition behaviour.

### 1.3. The Present Study

In recent years, some studies have focused on the importance of emotions and motivations during PE, showing that both psychological factors play important roles predicting the intention to be physically active [17,18,41]. Some of these studies have showed that not all the emotions and motivational regulations played the same role. For that reason, firstly, we wanted to know what the variables are in each construct with the highest ability to predict the intention to be physically active. Secondly, from a cognitive perspective, it is not yet known how the motivation and emotion experienced by adolescents in PE classes may be conditioned by the amount of extracurricular PA they perform. Recently, Zhang et al. [42] highlighted that student’s ball skill competence predicted enjoyment of PA. However, they did not study whether students with high levels of ball skills were the same students with high enjoyment and level of PA. This fact could be because students with higher physical performance are those who perform more hours of extracurricular PA [43]. Therefore, we want to know whether the psychological experience during PE is different based on the student’s extracurricular PA using cluster analysis. Specifically, profile-based analyses provide the ability to create taxonomies in which groups of individuals can be assigned to different individuals according to some criteria of homogeneity. Along this line of study, we find studies that have attempted to establish motivational profiles and their relationship with a greater amount of physical sport exercise practised [44,45,46], even after completing compulsory education [47,48], and more positive psychological consequences [49]. However, few studies have used constructs relating to motivational and emotional profiles together in the same sense at other educational stages [50]. To this end, in this study we propose as its first objective finding the level of PA and comparing it to the recommendations. Based on the above research, it is hypothesised that students are still less physically active than recommended (H1). Secondly, considering the large number of variables that can influence the intention to be physically active, the second objective of the study was to find which variables are the most significant. To this end, BPNs (autonomy, competence, and relatedness), motivation (autonomous, controlled, and amotivation), and emotions (pride, enjoyment, anger, anxiety, hopelessness, and boredom) were considered as predictors of the intention to be physically active. Based on the above-mentioned results, we hypothesised that competence, autonomous motivation, and enjoyment would be the variables with the highest predictive power of the intention to be physically active (H2). Given this information, the main aim of the study was to evaluate the differences between these most important variables in predicting the intention to be physically active according to each PA level’s profile. We postulate that the practice of extracurricular PA is highly correlated with the experience that students have in their PE classes. Thus, students with a high rate of participation in extracurricular PA will have a better experience during classes and, therefore, a greater desire to continue practising PA outside of school (H3). The results can help us to better understand what the behaviour of each variable is depending on the PA levels, and with this information we can propose different strategies to make PE lessons more satisfying from a psychological point of view, which will result in adolescents being more active.

## 2. Methods

### 2.1. Participants

Participants were 811 secondary school students (371 boys and 440 girls) from five secondary schools (public and private) in Huelva (Spain), aged between 11 and 17 years (M = 13.15, SD = 1.16). Each secondary school had students from 1st (*n* = 261), 2nd (*n* = 284), 3rd (*n* = 207), and 4th years (*n* = 59) of compulsory secondary education. Students take PE each year (2 h a week). The selection of the sample was performed by choosing the students in those secondary schools who agreed to participate in the study (non-probabilistic).

### 2.2. Measures

#### 2.2.1. Habitual Physical Activity

The students self-reported a series of ad hoc questions to determine their PA level. The first question was “Do you practice any sport or physical activity outside school?”. Students who did practise PA were asked about the type of PA or sport. Finally, they had to respond with the number of hours they practised each day. From this score, the number of hours per week for each subject was calculated.

#### 2.2.2. Physical Activity Intentions

Students’ perceptions of intention to be physically active were evaluated with the Spanish version [51] of the Intention to be Physically Active Scale [21]. This scale consists of 5 items (e.g., “I am interested in developing my physical fitness”) preceded by the stem “Regarding my intention to practice sport…”. The items are answered on a 5-point Likert-type scale ranging from 1 (strongly disagree) to 5 (strongly agree). Evidence for the reliability and validity of this questionnaire has been provided in the PE context [18,52]. Cronbach’s Alpha and McDonald’s Omega value were α = 0.78 and ω = 0.89.

#### 2.2.3. Satisfaction of Basic Psychological Needs

BPNs were evaluated using the scale developed by Moreno-Murcia et al. [53]. The scale is composed of three dimensions: autonomy, 4 items (e.g., “I feel very strongly that I have the opportunity to make choices with respect to the way I exercise”); competence, 4 items (e.g., “I feel that exercise is an activity in which I do very well”); and relatedness, 4 items (e.g., “I feel very much at ease with the other exercise participants”). The scale is introduced by the statement “In my physical education class…” and the answers are on a Likert scale ranging from 1 (strongly disagree) to 5 (strongly agree). Previous studies have tested this instrument’s reliability and validity [54,55]. Cronbach’s Alpha and McDonald’s Omega value were α = 0.78 and ω = 0.79 for autonomy; α = 0.77 and ω = 0.78 for competence; α = 0.83 and ω = 0.83 for relatedness, respectively.

#### 2.2.4. Motivational Regulations

To evaluate the regulation of behaviours in PE classes, the Spanish version [56] of the Perceived Locus of Causality Scale [57] was used. The scale features 24 items grouped among 6 factors that measure intrinsic motivation (e.g., “Because physical education is fun”), integrated regulation (e.g., “Because I consider physical education to be a part of me”), identified regulation (e.g., “Because I want to learn sports skills”), introjected regulation (e.g., “Because I would feel bad about myself if I did not), external regulation (e.g., “Because I will get into trouble if I do not”), and amotivation (e.g., “But I really do not know why”). The scale begins with the stem “I take part in physical education…” and the items are answered on a 7-point Likert-type scale ranging from 1 (strongly disagree) to 7 (strongly agree). Based on SDT and previous studies in PE the different regulations were grouped into autonomous motivation (intrinsic, integrated, and identified regulations), controlled motivation (introjected and external regulation), and amotivation [58,59]. Cronbach’s Alpha and McDonald’s Omega value were satisfactory (autonomous α = 0.94, ω = 0.94; controlled motivation α = 0.78, ω = 0.78; and amotivation α = 0.75, ω = 0.74).

#### 2.2.5. Achievement Emotions Questionnaire for Physical Education

The original version of the AEQ-PE was used [35]. This scale, validated with Spanish students, comprises 24 items divided into 6 emotions (4 for each emotion) covering the three main quadrants of the CVTAE [34]. The emotions measured are: pride (e.g., “I am proud of my participation in physical education class”), enjoyment (e.g., “I enjoy being in the physical education class”), anger (e.g., “I feel anger welling up in me during the physical education class”), anxiety (e.g., “I feel nervous in the physical education class”), hopelessness (e.g., “It is pointless to prepare for the physical education class because I am bad at it anyway”), and boredom (e.g., “I get bored during the physical education class”). Participants indicate the extent to which they agree with the statements on a 5-point scale (1 = totally disagree and 5 = totally agree). Evidence for the reliability and validity of this questionnaire has been provided in the PE context [18,60]. Cronbach’s Alpha and McDonald’s Omega value were autonomy α = 0.81 and ω = 0.82; enjoyment α = 0.84 and ω = 0.84; anger α = 0.79 and ω = 0.79; anxiety α = 0.80 and ω = 0.80; hopelessness α = 0.80 and ω = 0.80; boredom α = 0.83 and ω = 0.84.

### 2.3. Procedure

Participation in the study was solicited through direct contact with school administrators and school boards. To that end, they were informed, and their cooperation was requested. Five of the secondary schools agreed to participate. As the students were minors, written authorisation was requested from both the school and the parents of the participants. The measurement instrument was administered by at least one member of the research group, who informed the participants about how to respond, reminded them that participation was anonymous, and requested honesty in their responses. The questionnaires were completed by the students in a quiet atmosphere that enabled them to concentrate on the task and averaged about twenty minutes to complete. The questionnaires were collected individually to verify that they had been completed correctly. This study was approved by the Andalusian (Spain) Ethics Committee for Biomedical Research (TD-OCME-2018), and it was conducted in accordance with the ethical principles of the American Psychological Association [61].

### 2.4. Data Analysis

This research had a non-experimental, quantitative, correlational, and cross-sectional design. Firstly, the reliability of the scales used was tested by Cronbach’s Alpha and McDonald’s Omega [62]. Secondly, some preliminary results were calculated focusing on descriptive statistics (mean and standard deviation). Third, to determine the univariate relationships among all the variables, we performed a correlation matrix of the variables calculating Pearson. Finally, to investigate the predictors of intention to be physically active, all the independent variables were included in multiple linear regression analyses by constructs (emotions, BPNs, and motivations). The standardised β coefficients for each variable and the regression diagnostics (collinearity: tolerance > 0.2 or variance inflation factor; VIF < 4 [63]) in the model were calculated.

Secondly, cluster analyses were conducted to examine whether subgroups could be defined based on students’ extracurricular PA levels. First, the scores of the hours of PA were standardised. To reduce the impact of univariate outliers, values more than 3 SD above or below the mean were removed [64]. The analysis required two steps, thereby combining hierarchical and non-hierarchical clustering methods [65]. First, a hierarchical cluster analysis was carried out using Ward’s hierarchical clustering method based on squared Euclidean distances. The hierarchical method was used as a preliminary step in identifying the cluster solutions, which then provided the input for the non-hierarchical procedures. Based on the hours of PA, two, three, four, and five cluster solutions were considered. Only cluster solutions which explained at least 50% of the variance were retained for the following step [66]. In the following step, the extracted initial cluster centres based on Ward’s hierarchical method were used as non-random starting points in an iterative, non-hierarchical k-means clustering procedure. To examine the stability of the remaining cluster solutions, a double-split cross-validation procedure was implemented by randomly splitting the total sample into two halves and applying the two-step procedure (Ward and k-means) to each subsample [67]. Next, the students in each half-sample were assigned to new clusters based on their Euclidean distances to the cluster centres of the other half of the sample. Then, through Cohen’s kappa (*K*), the means of both clusters were compared. The two resulting *K* were averaged and an average *K* of at least 0.60 (good agreement) was considered acceptable [68]. In order to detect possible variables that could affect the results, the chi-square was calculated to see the sex and age distributions in the clusters.

Finally, once the students were divided into groups by PA hours, and once the most important variables for predicting the IPA were known, MANCOVA analyses and post-hoc (Bonferroni) comparisons were conducted to find the difference in these variables for each cluster. Effect sizes (partial eta-squared; *η_p_^2^*) above 0.01 were considered small, above 0.06 moderate, and above 0.14 large [69]. All the analyses were performed using SPSS 23.0 (IBM Corp., Armonk, NY, USA).

## 3. Results

### 3.1. Descriptive Statistics, Correlations, and Regression Analyses

The descriptive results of the practice of extracurricular PA showed that around 29% (*n* = 237) of the students did not practise PA. At the 50th percentile were the students who practiced for 3 h and at the 75th percentile were the students who practised for 5 h. Other descriptive statistics and correlations between the study variables are given in Table 1. For example, positive emotions (pride and enjoyment), basic psychological needs, and autonomous motivation were moderate–large, and positively and statistically correlated between themselves and between intention to be physically active and PA. On the other hand, negative emotions (i.e., anger, anxiety, hopelessness, and boredom), were moderate–large statistically and positively correlated with themselves (except the relationship between anxiety and boredom which was small), and statistically and negatively correlated with the rest of the variables. Specifically, hopelessness and anger had moderate–large relationships with the rest of the variables, and anxiety and boredom had small–moderate relationships. Controlled motivation was the only variable which did not correlate with most of the other variables.

To select the most important variables in each construct predicting the intention to be physically active, regression analyses were performed including emotions, BPNs, and motivations as predictors of the IPA (Table 2). Emotions explained 25% of the intention to be physically active (*F* _(6793)_ = 44.20; *p* < 0.00) with pride, enjoyment, and hopelessness being statistically significant. BPNs explained 30% of the intention to be physically active (*F* _(6796)_ = 111.75; *p* < 0.00), with competence the only statistically significant BPN. Finally, motivations explained 36% of the intention to be active (*F* _(6796)_ = 151.56; *p* < 0.00), with autonomous motivation being statistically significant.

### 3.2. Cluster Results: Physical Activity Profiles

**Figure 1:**
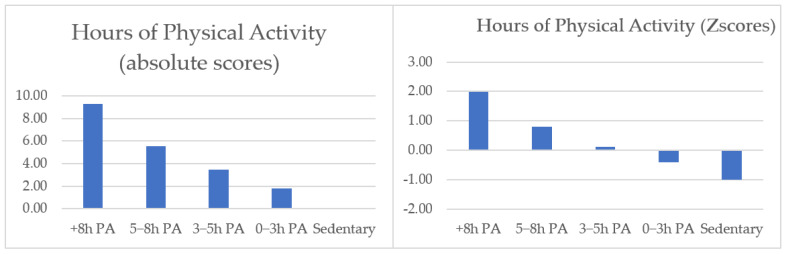
Five-cluster solution based on Z-scores and absolute scores for physical activity levels.

Before conducting cluster analyses, 11 univariate outliers were removed, resulting in a final sample of 800 students. Next, to identify the clusters, hierarchical and non-hierarchical clustering procedures were conducted. The two-cluster solution explained 64% of the variance in the level of PA, dividing the sample into the students who practise < 5 h (*n* = 599; 75%) or > 5 h (*n* = 201; 25%). The three-cluster solution explained 82% of the variance in the PA levels, classifying students into PA < 1 h (*n* = 264; 33%), 1 h > PA < 5 h (*n* = 335; 42%), or PA > 5 h (*n* = 201; 25%) per week. The four-cluster solution explained 91% of the variance explained, dividing the students into PA < 1 h (*n* = 264; 33%), 1 h > PA < 5 h (*n* = 335; 42%), 5 h > PA < 8 h (*n* = 139; 17%), or PA > 8 h (*n* = 62; 8%). Finally, the five-cluster solution explained 95% of the variance and divided the sample into 0 h called “sedentary” (*n* = 237, 30%), 0 h > PA < 3 h called “0–3 h PA” (*n* = 152; 19%), 3 h > PA < 5 h called “3–5 h PA” (*n* = 210; 26%), 5 h > PA < 8 h called “5–8 h PA” (*n* = 139, 17%), or PA > 8 h called “+8 h PA” (*n* = 62; 8%). Based on these results and taking into account the number of students who never practise PA in their free time (*n* = 237, 30%), the results of the dendrogram, and the agglomeration coefficient, we decided to work with the five-cluster solution. The stability of the cluster was good in each half (*K* < 0.90). In Figure 1, the levels of PA are presented as Z-scores (right half) and absolute scores (left half), showing statistical differences between all the groups (F _(4795)_ = 4429.32; *p* < 0.001; *η_p_^2^* = 0.96). The chi-square test revealed a non-significant cluster assignment by age (*χ*^2^ (24) = 31.27, *p* = 0.15) and significant cluster assignment by gender (*χ*^2^ (4) = 63.38, *p* = 0.00), showing that students were not well distributed. Therefore, gender was added as a covariate in further analyses.

### 3.3. Differential Analysis

**Figure 2:**
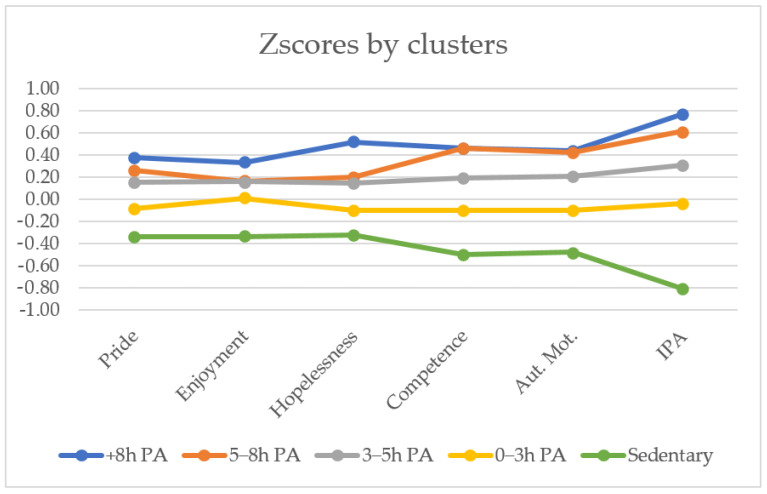
Clusters (all scores are standardised). Hopelessness scores have been recoded. PA, physical activity. IPA, intention to be physically active.

A MANCOVA (Table 3) was conducted to identify the characteristics of each cluster on the variables selected. Due to the results of Box’s Test, *M* = 349.73, *F* [112; 313467.12] = 3.05, *p* < 0.00 (violation of the homogeneity of covariance), Pillai’s Trace was used as a statistical test. Results (Table 3) indicate significant differences between the clusters Pillai’s Trace = 0.92, *F* (7, 788.00) = 1340.82, *p* < 0.00, and *η_p_^2^* = 0.923. For example, profiles with high levels of PA displayed higher positive emotions (pride and enjoyment) (small effect size), lower negative emotion (hopelessness) (small effect size), and higher competence, autonomous motivation, and intention to be physically active (moderate and large effect sizes) (Figure 2).

Post hoc analyses revealed a differentiation between clusters in which PA was over 3 h and clusters in which PA was under 3 h for pride and hopelessness. Similar results were found for enjoyment, except that the main difference was between sedentary cluster 5 and the rest of the clusters, while the results obtained for clusters in which PA was over 3 h were similar among them and different to clusters in which PA was under 3 h and sedentary cluster 5. As for pride and hopelessness, for competence and autonomous motivation a differentiation between clusters over and under 3 h of PA can be observed; moreover, statistical differences were found between the cluster with PA under 3 h and the sedentary cluster. Finally, focusing on the intention to be physically active, clusters in which PA was over 5 h presented similar results among them and different to clusters in which PA was under 5 h, which in addition, were different to each other (Table 3).

## 4. Discussion

The main findings of this study suggest that the PA levels are highly correlated with the student psychological experience during PE classes, finding that the profiles that perform more hours of extracurricular PA have a better psychological experience in the classes. Various previous studies have highlighted the importance of students’ perception of PE classes as a satisfying experience. This will have a number of positive consequences on their lives, such as increased psychological well-being [70] and increased intention to be physically active in the future [17], reflecting better physical and mental health [4,6,7,8]. For this reason, knowing all the variables which could affect the PE experience should be a priority for teachers and researchers. Based on this approach, in the present study, we wanted to verify the degree to which PA levels could be related with the students’ experience during PE classes. To this end, first we proposed finding the PA levels of the students and comparing them to the recommendations established by the reference organisations. Based on previous studies, it was hypothesised that students continue to practise less PA than is recommended (H1). The results obtained are similar to previous studies with Spaniards [71], finding that around 30% of students do not practise extracurricular PA. Taking into account that PE classes were for 1 h per day, 2 days a week, around 75% of the population did not achieve the recommendations of 1 h of PA per day set by the WHO [10].

Reducing this highly sedentary lifestyle and inactivity among children and adolescents should be a priority for the education system given the aforementioned impact on health. In this sense, PE plays a decisive role as many studies have shown so far [72]. PE is the only PA that all youth are guaranteed to perform [73], thus, PE teachers should try to make this experience a positive one and take advantage of the opportunity to encourage adolescents to engage in extracurricular PA [74,75]. Some of the variables that have been shown to indicate whether this experience is positive or not are the emotions and motivation students feel about their classes [76]. Thus, students’ motivation and emotional experience have been shown to have consequences for students’ behaviours [77], among which the intention to practise PA in the future stands out (e.g., [16,18]). Specifically, in the present study, we found that enjoyment, pride, and hopelessness were the emotions that had the greatest predictive power on intention to be physically active. Although enjoyment was the emotion with the greatest predictive power, as had been hypothesised (H2) and found in previous studies [38,39], by expanding the range of emotions studied, other emotions were found that also play an important role, in line with recent research [18]. In turn, both autonomous motivation and the psychological need for competence stood out as the variables capable of explaining the highest percentage of intention to be physically active (H2), as occurred in previous studies [52,74].

Knowing the most determining variables for intention to be physically active and the high variability in PA levels, the present study wanted to evaluate how this large difference in students’ PA levels might be related with their experience in PE classes. It was hypothesised that students who practised more hours of extracurricular PA were also those who had the best experience during PE classes and, in turn, those who were most likely to remain active (H3). The results obtained confirmed this assumption, as the groups that practised more hours of PA gradually obtained better experience in their PE classes and higher levels of intention to be physically active in the future. Despite this trend (more PA hours, better experience in PE classes), the cluster analysis based on PA levels showed that beyond certain weekly PA hours, the experience in PE classes did not vary. This cut-off point has been established mainly at two levels. The first one is between the sedentary groups and those who practise some physical activity, even if it is only for a short time per week (variables such as enjoyment, competence, and autonomous motivation). The second one is made up of students who practised more or less than 3 h of extracurricular PA (variables such as pride, competence, or autonomous motivation). In other words, the emotional and motivational experience is satisfactory and similar in the groups that practised at least 3 h of extracurricular PA, which added to the mandatory 2 h of PE per week, making a total of 5 h of PA per week.

The number of hours of extracurricular PA is strongly associated with the degree of motor competence of the students [43]. Thus, increasing the number of hours of PE to a mandatory 1 h per day could be a considerable change that would help to equalise the experience of students during PE classes. Spain only devotes an average of 53 h per year to PE (3% of total teaching hours) compared to neighbouring countries such as France, which devotes 144 h (14% of the total) [14]. In this way, on the one hand, an increase in the number of hours of practice could be guaranteed, approaching the WHO recommendations [10]. On the other hand, by increasing the hours, an improvement in the experience during classes would be expected, especially in the groups with the good perception, due to the improvement in their motor skills [43]. This fact would have both a direct and an indirect consequence on the intention to be physically active. Along these lines, in other countries such as the United States, they are trying to apply intervention programs such as the Comprehensive School PA Program [78]. This program, based on five main components (PE, PA during school, PA before and after school, staff involvement, and family and community engagement), tries to enable students to accumulate a minimum of 1 h PA per day and develop knowledge, skills, and confidence for a lifelong PA style.

Consequently, students who perform little or no extracurricular PA (around 50% of the study population) have a less good experience during their classes and a lower intention to be active. This fact represents a dangerous trend for the health of future adults, since if we do not reverse this situation, these students may continue to be sedentary all their lives [3]. Since PE and PE teachers play a determinant role in the promotion of active habits [79], one of the priority lines of action could be aimed at increasing the number of hours of PE, trying to equalise the levels from primary education in other European countries [11], as has been proposed earlier. In turn, it is recommended that teachers try to improve the physical experience of those students who practise the least. These students are possibly the least physically developed [14] and the ones who most need to be encouraged to perform PA outside of school. In this sense, pedagogical models (e.g., sport education, cooperative learning, social responsibility, etc.) have been shown to influence the perception of some psychological variables. It is also recommended that these strategies be in line with established theory such as SDT [28,31] or CVTAE [34], since they have shown to be effective in improving the experience during PE classes [16,18,36,80]. For example, teachers could involve students in the design of tasks or encourage autonomous behaviour to satisfy autonomy; they could provide positive feedback to satisfy competence; and they also could be friendly and attentive to their students to satisfy relatedness [81]. Moreover, related to novelty, PE teachers could propose a wide and innovative range of PA during classes away from conventional sports [55], thus offering students possibilities to experience new forms of PA in environments where they feel comfortable and responsible for their own actions, which will increase their enjoyment [82]. On the other hand, developing projects that show the diversity of extracurricular practise possibilities offered by the environments could help encourage students to start new practices. Other strategies that teachers could apply to improve the emotional experience focused on the CVTAE could be to use inclusive and cooperative environments to generate enjoyment; to avoid comparison between schoolmates in order not to generate hopelessness; or to make students experience success to feel pride.

Despite the interesting results obtained, and the possible practical applications suggested, the results should be taken with caution since this is a correlational and cross-sectional study. Experimental and longitudinal studies examining the phenomenon described above could help provide a deeper understanding of the problem and could contribute to finding effective measures to solve it. Another limitation of this study is that the PA level variable was created based on self-reported duration and frequency and did not take into account the intensity of the PA. It would be interesting in the future to use objectively measured PA, to add other variables related to PA, and to include the level of motor development of the students.

## 5. Conclusions

The results of the study have shown that the hours of extracurricular PA have a strong correlation with students’ emotional and motivational experience of their PE classes. In turn, this is reflected in the intention to be active in the future. Based on these findings, a series of strategies are proposed at both the institutional level (e.g., increasing the number of PE hours, apply interdisciplinary programs, etc.) and the teacher level (improving the practical experience of the groups that practise less non-academically, offering a wide range of possibilities for extracurricular practice, etc.). These proposals are intended to improve the motivational and emotional experience during their PE classes of students who engage in less extracurricular PA. It could also be interesting to apply and investigate these ideas in extracurricular activities to encourage this habit in adolescents.

## Figures and Tables

**Table 1:** Descriptive statistics and bivariate correlations.

Variables	1	2	3	4	5	6	7	8	9	10	11	12	13	14
1. Pride	-	0.71 **	−0.40 **	−0.25 **	−0.51 **	−0.51 **	0.95 **	0.43 **	0.63 **	0.62 **	0.06	−0.39 **	0.43 **	0.25 **
2. Enjoyment		-	−0.48 **	−0.22 **	−0.56 **	−0.68 **	0.58 **	0.47 **	0.61 **	0.73 **	0.01	−0.48 **	0.45 **	0.20 **
3. Anger			-	0.33 **	0.52 **	0.57 **	−0.31 **	−0.38 **	−0.37 **	−0.39 **	0.07	0.47 **	−0.23 **	−0.14 **
4. Anxiety				-	0.46 **	0.20 **	−0.17 **	−0.33 **	−0.35 **	−0.20 **	0.14 *	0.27 **	−0.17 **	−0.19 **
5. Hopelessness					-	0.49 **	−0.33 **	−0.38 **	−0.57 **	−0.50 **	−0.02	0.52 **	−0.38 **	−0.25 **
6. Boredom						-	−0.48 **	−0.38 **	−0.41 **	−0.56 **	0.05	0.47 **	−0.28 **	−0.13
7. Autonomy							-	0.38 **	0.57 **	0.61 **	0.04	−0.36 **	0.35 **	0.15 **
8. Competence								-	0.54 **	0.48 **	0.00	−0.32 **	0.34 **	0.19 **
9. Relatedness									-	0.66 **	0.08 *	−0.41 **	0.54 **	0.34 **
10. Autonomous Motiv.										-	0.18 *	−0.51 **	0.60 **	0.32 **
11. Controlled Motiv.											-	0.28 **	0.11 **	0.07 *
12. Amotivation												-	−0.32 **	−0.22 **
13. IPA													-	0.53 **
14. Physical Activity														-
Range	1–5	1–5	1–5	1–5	1–5	1–5	1–5	1–5	1–5	1–7	1−7	1−7	1−5	-
Mean	3.97	4.03	1.46	2.00	1.45	1.85	3.04	4.20	3.79	5.36	3.96	2.37	4.19	2.93
Standard Deviation	0.88	0.91	0.69	0.87	0.69	0.90	0.90	0.86	0.86	1.28	1.21	1.33	0.82	2.74

Note. IPA = intention to be physically active; * = *p* < 0.05; ** = *p* < 0.01.

**Table 2:** Results of regression analyses.

Variables	*R* ^2^	*β* (BCa 95% CI’s)	T	*p*	Tolerance	VIF
**IPA**	0.25					
1. Pride		0.19 (0.09, 0.26)	4.25	0.00	0.48	2.06
2. Enjoyment		0.30 (0.17, 0.36)	5.63	0.00	0.34	2.93
3. Anger		0.04 (−0.05, 0.14)	1.02	0.31	0.58	1.73
4. Anxiety		−0.01 (−0.07, 0.06)	−0.25	0.76	0.76	1.31
5. Hopelessness		−0.18 (−0.31, −0.11)	−4.11	0.00	0.51	1.95
6. Boredom		0.08 (−0.01, 0.16)	1.85	0.07	0.45	2.21
**IPA**	0.30					
7. Autonomy		0.06 (−0.01, 0.12)	1.59	0.11	0.67	1.49
8. Competence		0.47 (0.37, 0.52)	11.79	0.00	0.55	1.80
9. Relatedness		−0.07 (−0.00, 0.13)	1.84	0.07	0.70	1.44
**IPA**	0.36					
10. Autonomous motiv.		0.59 (0.33, 0.42)	16.39	0.00	0.63	1.60
11. Controlled motiv.		0.02 (−0.03, 0.05)	0.55	0.58	0.78	1.28
12. Amotivation		−0.03 (−0.06, −0.03)	−0.73	0.47	0.59	1.68

Notes: IPA, intention to be physically active; *R*^2^, coefficients of determination; *β*, standardised regression coefficient; (BCa 95% CI’s), bias-corrected accelerated 95% confidence intervals; VIF, variance inflation factor.

**Table 3:** Comparison of cluster means, standard deviations, z scores for the clustering and MANCOVA results.

	Cluster 1 (a) +8 h PA			Cluster 2 (b) 5–8 h PA			Cluster 3 (c) 3–5 h PA			Cluster 4 (d) 0–3 h PA			Cluster 5 (e) Sedentary			*F*-Value	*η_p_* ^2^
	*N* = 62 (7.75%)			*N* = 139 (17.38%)			*N* = 210 (26.25%)			*N* = 152 (19%)			*N* = 237 (29.63%)				
	M	SD	Z	M	SD	Z	M	SD	Z	M	SD	Z		SD	Z		
1. Physical activity level	9.27 *	1.37	2.00	5.55 *	0.70	0.79	3.44 *	0.50	0.11	1.82 *	0.38	−0.41	0.00 *	0.00	−1.00	4429.32	0.96
2. Pride	4.31 ^d,e^	0.86	0.38	4.20 ^d,e^	0.78	0.26	4.11 ^e^	0.81	0.16	3.90 ^a,b^	0.82	−0.08	3.68 ^a,b,c^	0.93	−0.34	13.78	0.07
3. Enjoyment	4.33 ^e^	0.76	0.33	4.18 ^e^	0.84	0.16	4.18 ^e^	0.86	0.16	4.04 ^e^	0.84	0.01	3.72 *	1.00	−0.34	11.28	0.05
4. Hopelessness	1.10 ^d,e^	0.24	−0.52	1.31 ^e^	0.63	−0.20	1.35 ^e^	0.62	−0.15	1.52 ^a^	0.68	0.10	1.67 ^a,b,c^	0.78	0.32	14.27	0.07
5. Competence	4.19 ^d,e^	0.81	0.46	4.19 ^d,e^	0.74	0.46	3.95 ^e^	0.75	0.19	3.71 ^a,b,e^	0.78	−0.10	3.36 *	0.88	−0.50	31.91	0.14
6. Autonomous motivation	5.92 ^d,e^	1.15	0.44	5.90 ^d,e^	1.09	0.42	5.63 ^e^	1.11	0.21	5.23 ^a,b,e^	1.16	−0.10	4.75 *	1.36	−0.48	29.10	0.13
7. IPA	4.82 ^c,d,e^	0.33	0.77	4.69 ^c,d,e^	0.53	0.61	4.44 *	0.53	0.31	4.16 *	0.74	−0.04	3.52 *	0.84	−0.81	99.25	0.33

Note. ^a–e^ Each letter shows the significant difference with the comparison group. The letter of each group is set in the first row next to the cluster number; *, these values are significantly different from all groups. IPA = intention to be physically active. All analyses were controlled for gender.
